# Response to: Comment on “The Effect of Cochlear Size on Cochlear Implantation Outcomes”

**DOI:** 10.1155/2021/6294914

**Published:** 2021-03-24

**Authors:** Jafri Kuthubutheen, Amandeep Grewal, Sean Symons, Julian Nedzelski, David Shipp, Vincent Lin, Joseph Chen

**Affiliations:** ^1^School of Surgery, University of Western Australia, Perth, Australia; ^2^Sunnybrook Health Sciences Centre, Toronto, Canada M4N3M5; ^3^University of Toronto, Toronto, Canada M5S 1A1

We thank the authors Eser et al. for their comments [1] on our manuscript entitled “The Effect of Cochlear Size on Cochlear Implantation Outcomes” [2]. We agree that the effect of cochlear size on cochlear outcomes is very topical and that much research still needs to be done in order to determine the relationship between the two variables.

As we discussed in our paper, there are a number of possible explanations for the correlation between cochlear size and speech outcomes only seen with the shorter electrode. These include factors related to insertion trauma and postoperative electrode movement. We also do not believe that absolute spiral ganglion counts or tonotopy is the reason for this observed correlation. However, this does not mean that these are not factors contributing to this correlation. For example, spiral ganglion *distribution* rather than absolute spiral ganglion counts may be important, particularly in those patients with better residual hearing function (preoperative consonant-nucleus-consonant (CNC) word score) which was the case in our group who received the shorter electrode.

We decided in our study not to measure the apical turn of the cochlea because we felt that the challenges of obtaining an accurate result using our described technique would outweigh the benefits of obtaining this data. We agree that understanding the apical anatomy of the cochlea is important, particularly when using lateral wall electrodes.

The authors correctly pointed out the error in Table 3 where the values of the outer wall length to 360 degrees should actually be those for the outer wall length to 720 degrees and vice versa. This has now been corrected [3].

The *p* values can be derived statistically from the correlation coefficient and the number of pairs. In this case, there are 21 pairs of data for the Flex 28 group. Therefore, the correlation between Ac and CNC (%) is 0.64 which has a *p* value of 0.00178, and the correlation between outer wall length and CNC (%) is 0.71 which has a *p* value of 0.000311. Similarly, the correlation between Ac and AZBIO (%) is 0.46 which has a *p* value of 0.03589, and the correlation between outer wall length and AZBIO (%) is 0.47 which has a *p* value of 0.031561.
